# From Precious to Earth-Abundant Metallic Nanoparticles: A Trend of Interband Transitions in Photocatalyzed Nitrobenzene Reduction

**DOI:** 10.1021/acs.jpcc.4c03940

**Published:** 2024-08-22

**Authors:** Pin Lyu, Lauren Hoffman, Daniel Valenzuela Cahua, Son C. Nguyen

**Affiliations:** † Department of Chemistry and Biochemistry, University of California, Merced, 5200 North Lake Road, Merced, California 95343, United States; ‡ Department of Chemistry and Biochemistry, University of North Carolina, Asheville, 1 University Heights, Asheville, North Carolina 28804, United States

## Abstract

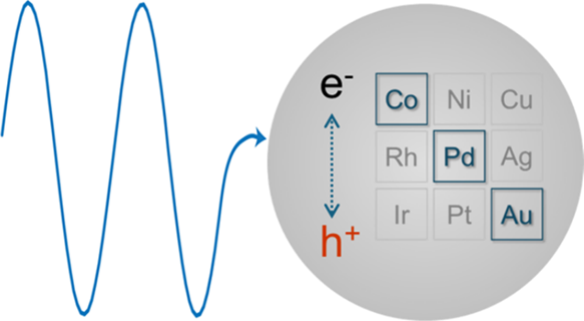

Metallic nanoparticles have been demonstrated to be versatile photocatalysts, as exemplified by those made from noble and precious metals. Transitioning from precious to earth-abundant metals for sustainable photocatalysis requires benchmarking their catalytic performance. In this work, we attempt to compare the photocatalytic activities of Au, Pd, and Co–B nanoparticles in the reduction of nitrobenzene by hydrazine. Despite their different morphologies and surface structures, Co–B nanoparticles offer the highest catalytic enhancement when comparing their reaction rates under irradiation to those under nonirradiation conditions. The trend of improved photocatalytic performance when transitioning from Au to Pd, and then to Co–B, can be explained by the nature of their d-band positions and corresponding hot carriers photogenerated from interband transitions.

## Introduction

Metallic nanocrystals have gained great interest in photocatalysis due to their strong and tunable light absorption, robust nature for multicycle operation, and versatile integration with other supporting materials.^1−13^ Currently, most metallic nanocrystal photocatalysts are based on noble metals, such as Au,^11,14,15^ Ag,^16−18^ Pt,^19,20^ and Pd.^13,21−23^ However, their high cost hinders large-scale applications for photocatalysis. Low-cost alternatives should be the non-noble and less precious versions.^24,25^ This study will explore the use of Co–B alloy nanoparticles and evaluate their potential for the transition from noble to more affordable materials in photocatalysis applications. The photocatalytic performance of the particles will be evaluated in a model reduction reaction, and the enhanced catalytic activities under photoexcitation will be compared with those of Au and Pd nanoparticle photocatalysts.

**Scheme 1:**
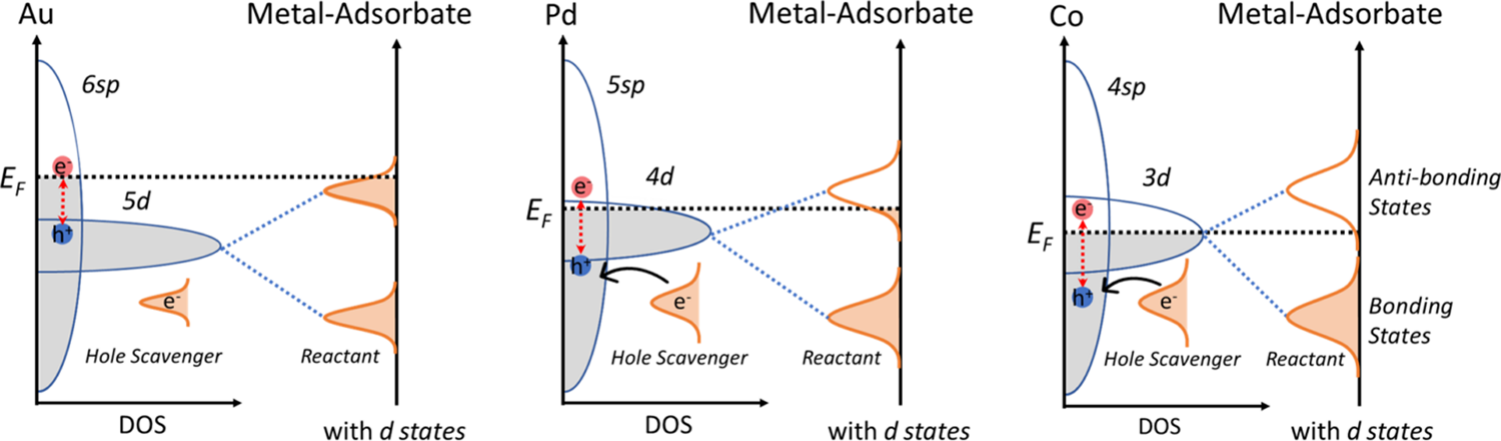
Comparison of Density of States (DOS), Interband Transitions (Generating Hot Electrons and Holes), and Metal–Adsorbate Splitting States (Bonding and Antibonding States) for the Three Studied Transition Metals (Au, Pd, and Co) The red arrows show some possible transitions in the metals. *E*_F_ is denoted as the Fermi level.

As we transition from noble to non-noble metals, moving from the bottom right to the top left of the periodic table, the nanoparticles composed of these metals have significant changes in the electronic and associated catalytic properties. Ideally, determination of d-band structures, density of states, and Fermi levels of these nanoparticles should be the starting points for our rationale for their catalytic performance. However, measuring these properties is beyond the scope of this report. Alternately, the trends in these changes can be predicted by leveraging our knowledge of solid-state chemistry, assuming that the particles are large enough to maintain metallic states. Particularly, the electronic interaction between the metals and catalyzed reactants can be roughly predicted from the valence electron energy levels of the metals. First, electron filling in the d-bands gradually shifts from full to partial when moving from noble to non-noble metals. According to the Newns–Anderson–Grimley model^26,27^ and d-band theory,^28−30^ the broad nature of the sp-bands across different transition metals leads to similar bond strength between metals and adsorbates, while less electron filling in d-bands results in less filling of metal–adsorbate antibonding states, and eventually strengthens the metal–adsorbate bonds (see Scheme 1). Second, the Fermi levels of these metals gradually shift from the sp- to d-bands, acquiring more d-character. Third, lower nuclear charge leads to less contraction of d-orbitals within individual atoms, but more d-orbital spatial overlap between neighboring atoms in the nanocrystals, which ultimately results in wider d-bands.^31^ The wider d-bands also give more d-character to the antibonding states of the metal–adsorbate states. All three factors, as illustrated in Scheme 1, contribute to stronger overlaps between d-orbitals of the metals and molecular orbitals of the adsorbates. This condition facilitates the adsorption of reactants on the metal surfaces, which is crucial for catalysis and often correlates with catalytic activities. For example, Co metal is a better catalyst for O_2_ dissociation than Pd and Au.^32^ Similarly, in the production of methane from syngas, Co offers a much higher turnover frequency than Pd and Au because it has stronger CO dissociative chemisorption, a rate-determining step in methanation.^28^ In the context of this study, Co–B alloyed nanoparticles are employed because they have similar catalytic properties as Co metal and they are less prone to oxidation. We expect that the reactants of the studied reactions have stronger adsorption on Co–B nanoparticles than on Au and Pd nanoparticles.

After the reactants adsorb on the metal surfaces, the photocatalysis process in metallic nanoparticles involves various mechanisms that facilitate energy and charge transfer, including hot-carrier generation and transfer,^33,34^ field enhancement,^35^ and photothermal effect.^36^ As for the photocatalyzed-reduction reaction demonstrated in this study, the first mechanism is our focus, and the properties of the photogenerated hot carriers depend strongly on the optical excitation regions and metal elements. As transitioning from noble to non-noble metals, the localized surface plasmon resonances (LSPRs) of the nanocrystals become less distinctive, but their interband transitions are ubiquitous and strong in the visible region.^37^ Unlike LSPRs, which can be tuned by varying the nanocrystals’ size, shape, and assembly, interband transitions are less influenced by these geometric factors. They depend primarily on the electronic structures of the metals, which makes their application adaptable to any size and morphology of metallic nanoparticles. Recently, interband transitions have been explored for producing “deep” hot holes below the Fermi levels, thereby enhancing catalytic activities of many reactions that benefit from the hot holes.^22,38−42^

Direct d-to-sp interband transitions in a late d-block metal like Au mostly generate hot electrons near the Fermi levels and hot holes well below the Fermi levels.^33,43,44^ As moving to Pd, then Co, the Fermi levels have more contribution of the d-shell, thus the interband transitions can generate more energetic electrons above the Fermi levels and the holes below the Fermi levels (see Scheme 1). In particular for Co, the interband transitions switch to an sp-to-d nature due to its different band structure.^45^ Our strategy for utilizing these carriers in catalyzing reduction reactions is quenching the hot holes by scavengers and accumulating electrons for catalysis. Given that the hot holes have a very short lifetime, in the order of tens of femtoseconds,^33,40,42,46^ quenching them with an abundant sacrifier is our strategy to enhance carrier extraction. The hot holes, possessing strong oxidation power, should be used to oxidize the sacrifier so that the hot electrons can either directly catalyze reduction reactions or be cooled, then modify the Fermi levels of the nanoparticles, and ultimately catalyze the same reactions. This photocharging mechanism, previously demonstrated for noble metal nanoparticles,^39,47^ leads us to hypothesize its applicability in utilizing interband transitions in non-noble metal nanoparticles. In this work, we relied on a photocharging mechanism to evaluate the photocatalytic performance of Co–B, Pd, and Au nanoparticles.

## Experimental Methods

### Chemicals and Characterizations

**Figure 1:**
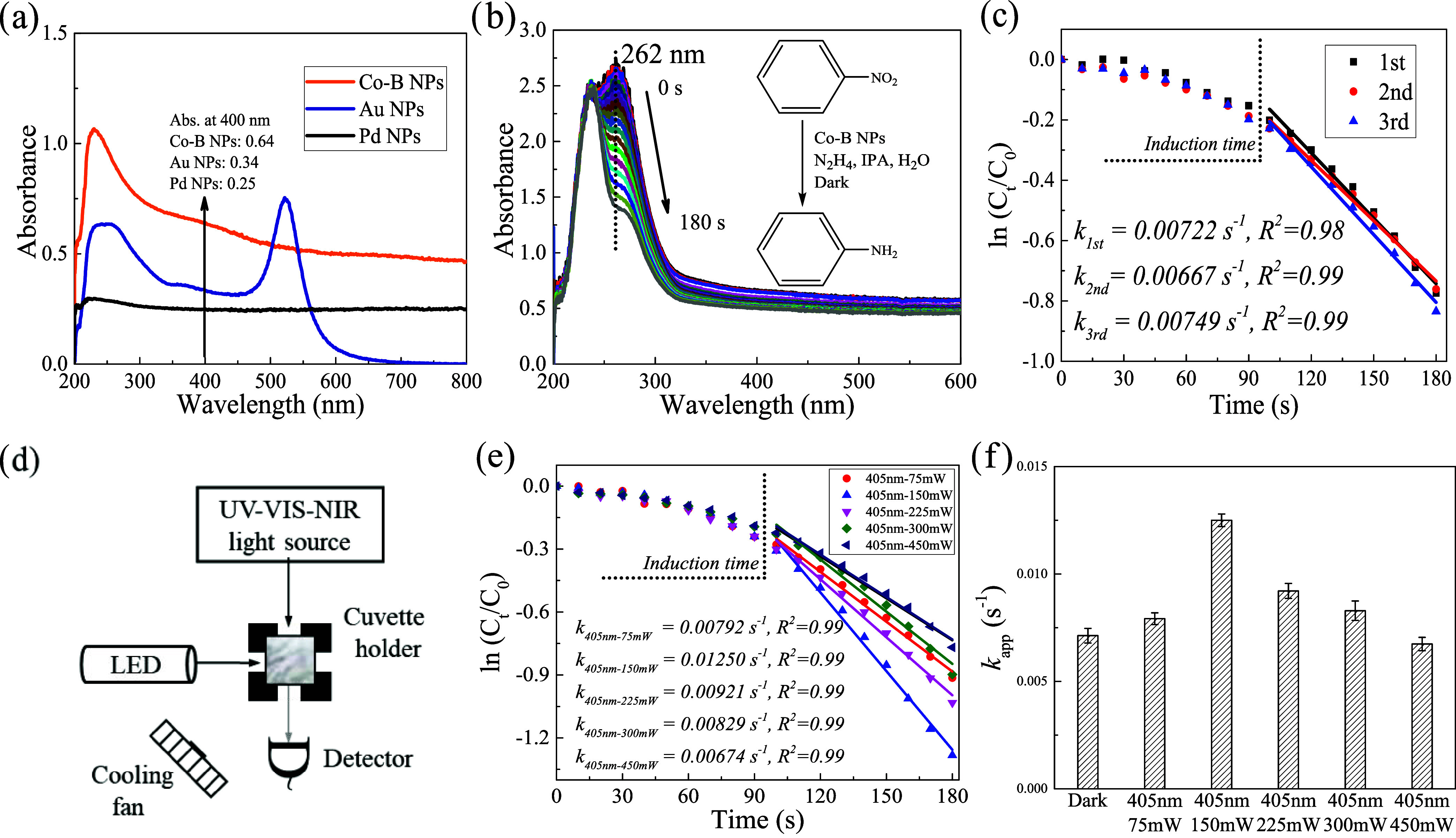
UV–vis spectra of three nanoparticle photocatalysts and nitrobenzene reduction catalyzed by Co–B nanoparticles. (a) UV–vis spectra of Co–B, Au, and Pd colloidal nanoparticles. The recorded amounts are the same as the catalyst loads in the reduction reaction. (b) UV–vis spectra of nitrobenzene reduction by N_2_H_4_ with Co–B nanoparticles under nonirradiation conditions. (c) Kinetic analysis for the three replicated reactions depicted in panel (b), the pseudo-first-order process for nitrobenzene, and the three replicated *k* values were extracted. (d) A setup for *in situ* monitoring kinetics of photocatalyzed reactions. (e) Similar kinetic analysis for the same reaction in panel (b) with 405 nm irradiation at various incident power. (f) Apparent reaction rate constants (*k*_app_) extracted from (c) and (e). All error bars represent one standard deviation of the mean.

All chemicals and reagents were used without any purification. Reaction kinetics were monitored by a modified UV–vis spectrometer (USB4000 Ocean Optics). As shown in Figure 1d, the cuvette holder of the spectrometer has an optical aperture that allows an light-emitting diode (LED) beam to be directed into the reaction solution in the cuvette. The cuvette holder was placed on top of a stir plate, and the reaction solution was constantly stirred. The morphology of Au, Pd, and Co–B nanoparticles were examined by transmission electron microscopy (TEM, Talos F200C G2, 200 kV, Thermo Fisher Scientific). The size distribution in the reaction solutions and ζ potential were measured by dynamic light scattering (DLS, Zetasizer Pro, Malvern Panalytical). The local crystalline structure of Co–B amorphous alloyed nanoparticles was determined by powder X-ray diffraction pattern (PXRD, PANalytical X’Pert PRO Theta/Theta, Co tubes, 40 kV, 45 mA) and was processed with X’Pert HighScore data analysis software. The surface oxidation state of Co–B nanoparticles used before and after catalyzed reactions was determined by X-ray photoelectron spectroscopy (XPS, Nexsa, Al Kα X-ray source, Thermo Fisher Scientific). The samples were prepared by drop-casting and drying the Co–B nanoparticle solution on a silicon wafer and sent for XPS immediately. The intermediates and products from nitrobenzene reduction were determined by proton nuclear magnetic resonance (^1^H NMR, Varian-INOVA 400 MHz, Agilent Technologies), where deuterated chloroform (CDCl_3_) was used as the solvent and mesitylene as the internal standard.

### Synthesis of Au Nanoparticles

The synthesis protocol was modified from the seed-mediated growth method developed by Xia’s group,^48^ and it was used in our previous work.^49^ Briefly, the Au clusters were first prepared by rapid injection of fresh NaBH_4_ solution (0.6 mL, 10 mM) into the 10 mL mixture of gold precursors HAuCl_4_ (0.25 mM) and cetyltrimethylammonium bromide (CTAB) (100 mM). After incubating for 3 h, the Au clusters (5 mL) were mixed with ascorbic acid (150 mL, 100 mM) and cetyltrimethylammonium chloride (CTAC) (200 mL, 200 mM) before the rapid injection of more precursors HAuCl_4_ (200 mL, 0.5 mM). Followed by incubation at 30 °C in 15 min and centrifugation, the 10 nm Au seeds were collected and grown to 38 ± 2 nm by dropwise adding more HAuCl_4_ precursors (200 mL, 0.5 mM), CTAC (200 mL, 100 mM), and ascorbic acid (1.3 mL, 10 mM). The final Au nanoparticles were washed and stored in 0.1 M CTAC solution for later use in photocatalysis.

### Synthesis of Porous Pd Nanoparticles

The synthesis protocol was modified from the hard-template method developed by Yamauchi’s group,^50^ and it was also used in our previous work.^22,47^ Briefly, polystyrene-*block*-poly(ethylene oxide) (PS_5000_-*b*-PEO_2200_, Polymer Source Co.) was used as the template (8 mg dissolved in 200 μL tetrahydrofuran (THF)), H_2_PdCl_4_ as the Pd precursor (500 μL, 76.8 mM), ascorbic acid (2 mL, 0.1 M) as the reducing agent, HCl for adjusting the pH (160 μL, 2 M), and finally incubating at 50 °C for 10 h. The final products were washed, calcined at 200 °C for 1 h to remove the excess polymers, and dispersed in water for later use in photocatalysis.

### Synthesis of Amorphous Alloyed Co–B Nanoparticles

The synthesis protocol was modified from the reduction method developed by Li’s group.^51^ Briefly, tetrabutylphosphonium bromide (51 mL, 0.05 M), cobalt(II) chloride (5.1 mL, 0.1 M), and KCl (21 g to form a saturated solution) were mixed well and then put into an ice bath for maintaining its temperature at 273 K. Then freshly made KBH_4_ (40 mL, 0.5 M) was injected into the mixture with a rate of 20 mL/h. After the reaction was complete, the black precipitates were washed and stored in ethanol solutions for further characterization and use in photocatalysis.

### Nitrobenzene Reduction with Co–B Nanoparticles

In a typical reaction condition, nitrobenzene (50 μL, 10 mM in isopropanol), isopropanol (950 μL, used as a solvent and hole scavenger), hydrazine hydrate (50 μL, 50–60% aqueous solution), Co–B nanoparticles (100 μL, 0.3 M based on Co element, stored in ethanol solution), and H_2_O (850 μL) were mixed in a four-clear-side quartz cuvette (1 × 1 cm^2^, R-3010-T, Spectrocell) and immediately underwent UV–vis spectra measurements with a time resolution of 1 s (see Figure 1d). The reaction solution in the cuvette was stirred as it was placed on top of a stir plate. The absorbance at 262 nm was assigned to nitrobenzene and corrected by subtracting the background absorbance of the Co–B nanoparticles. The corresponding concentration of nitrobenzene (*C*_t_) based on Beer’s law was fitted to the linear plot of ln(*C*_t_/*C*_0_) *vs* time to extract the apparent reaction rate constant *k*_app_.

As for the photocatalyzed reactions, LEDs with different emitting wavelengths (405, 415, 450, 490, 530, 595 nm, Thorlabs) were used to photoexcite the nanoparticles in the four-clear-sided cuvette, a cooling fan was used to keep the reaction at room temperature, and the other conditions remained the same with typical reaction condition above, unless specified. The incident power was measured by a power meter (PM100D console with S170C sensor, Thorlabs) and kept around 150 mW, except for experiments that needed to adjust the incident power. The 405 nm LED is the lowest wavelength light source in our experiment as it has no interference with the cutoff absorbance at 380 nm of nitrobenzene and aniline in the reaction solutions (Figure S10).

### Nitrobenzene Reduction with Au and Pd Nanoparticles

For Au nanoparticles, since the large amount of isopropanol in the reaction solution causes aggregation of the particles, the typical reaction condition was modified to maintain the colloidal form of the particles. Nitrobenzene (50 μL, 10 mM, isopropanol as solvent), hydrazine hydrate (50 μL, 50–60% aqueous solution), Au nanoparticles (500 μL, 0.26 mM based on Au element, stored in CTAC solution), and H_2_O (1400 μL) were mixed as the reaction solution. The following steps remained the same as in the case of Co–B nanoparticle catalysts.

For Pd nanoparticles, the only modification from the typical reaction conditions was the amount of H_2_O (700 μL) and Pd nanoparticles (250 μL, 2.26 mM based on Pd element, stored in water). Eventually, the metal concentration in the reaction solutions with Co–B nanoparticles was 27 times higher than that with Pd nanoparticles. The metal concentration in the reaction solutions with Pd nanoparticles was 8.7 times higher than that with Au nanoparticles.

### Nitrobenzene Reduction with Photocharged Co–B Nanoparticles

To prove the photocharging mechanism, our approach in a previous study is to separate the photocharging step from the catalysis step and to evaluate the catalytic performance with different charging conditions.^47^ The photocharging step was followed by irradiating the stock Co–B nanoparticles (0.3 M, based on Co element, stored in ethanol solution) with a 450 mW incident power of a 405 nm LED. Ethanol was the hole scavenger. After a certain amount of charging time (varying from 10 min to 9 h), 100 μL of photocharged Co–B nanoparticle solution was used as the catalyst for the nitrobenzene reduction in the dark. The rest of the reaction protocol and data analysis were conducted exactly as steps in photocatalyzed reactions.

## Results and Discussion

### Establishing Conditions for Comparing Photocatalytic Performance of Au, Pd, and Co–B Nanoparticles for Nitrobenzene Reduction

All the nanoparticles were synthesized, cleaned, and dispersed in colloidal form before being used as photocatalysts (Figure S1, and more details are given in the Experimental Methods section). Au nanocrystals were prepared in spherical form by the seed-mediated growth method, using HAuCl_4_, CTAB, and ascorbic acid as the precursor, capping ligand, and reducing agent, respectively.^49^ Pd nanocrystals were prepared in mesoporous form by the hard-template growth method using H_2_PdCl_4_, PS-*b*-PEO polymer, and ascorbic acid as the precursor, template, and reducing agent, respectively.^50^ Co–B amorphous alloyed nanoparticles were prepared by reducing CoCl_2_ with BH_4_^–^ in the presence of the Bu_4_P^+^ ligand.^51^ In the original study on preparing these Co–B particles, their thermodynamically metastable state was confirmed by differential scanning calorimetry; and the amorphous alloy structure was verified through transmission electron microscopy and X-ray diffraction. The metallic state of cobalt, as well as both elemental and oxidized states of boron, were confirmed by X-ray photoelectron spectroscopy. More importantly, catalysis of these Co–B nanoparticles under nonirradiation conditions showed the activity of cobalt metal, while boron did not significantly contribute to the catalytic mechanism.^51^

To ensure comparable photocatalytic performance among the nanoparticles of the three metals, 405 nm light-emitting diodes (LEDs) were used to excite their interband transitions. Au nanocrystals are known to have direct interband transitions around this wavelength.^40^ Our previous study showed that mesoporous Pd nanocrystals exhibit strong interband transitions around the 400 nm region, and their LSPRs shift toward the red-photon region.^22^ As for Co–B nanoparticles, their amorphous and alloyed structures are expected to impede the collective oscillation of the free electrons and suppress LSPR. We believe that their absorption at 405 nm is attributable to interband transitions, and the nearly flat absorbance observed around this region further supports this interpretation (Figure 1a). When interband transitions were selected for these three catalysts, we measured the reaction rates under both irradiation and dark conditions. The ratios of reaction rate constants under 405 nm irradiation to those in the dark serve as the most appropriate factors for evaluating the relative performance of these three catalysts. The enhancement factors allow for photocatalytic comparisons across the catalysts, despite their differences in their morphologies and surfaces.

The reduction of nitrobenzene by hydrazine is selected as our model reaction, as nitroarene reduction can be catalyzed by various metallic nanoparticle photocatalysts.^52−55^ Isopropanol was used as the hot-hole scavenger (see detailed reaction conditions in the Experimental Methods section). Due to the spectral overlap between nitrobenzene and aniline product (Figure S5), the kinetic trace at 262 nm for nitrobenzene bleaching is our best option to determine the reaction rate (Figure 1b). For Co–B nanoparticle catalysts, the reaction under nonirradiation (dark) conditions was reproduced three times to obtain a reliable induction time (90 s). Accordingly, the kinetic traces from 90 to 180 s were well-fitted to a pseudo-first-order process, which has been known for reduction of nitrobenzene and allowed for the extraction of the apparent reaction rate constants (*k*_app_) (Figure 1c).^56^ The reaction did not proceed without hydrazine or Co–B nanoparticles (Figure S6). The photocatalyzed reactions were conducted in the same cuvette used under dark conditions except that the LEDs were turned on and their optical power was adjusted (Figure 1d). The wavelengths of the light were selected to avoid the absorption of the reactants and products involved in nitrobenzene reduction (Figure S5). The nanoparticles are light absorbers. The UV–vis spectra of the reaction solutions in the cuvette were recorded every second, allowing sufficient measurement of their kinetics, even though the reaction was completed within a few minutes. Both UV–vis (Figure S7) and NMR (Figure S8) spectroscopies confirmed that the final reduction product was aniline, similar to the products observed under dark conditions. To extract the *k*_app_ under photocatalyzed conditions, the same induction time and linear fitting methods were applied as those under dark conditions.

It is important to consider any undesired effects that could interfere with the photocatalytic mechanism on which we focus on. First, the photothermal effect should not interfere with the hot-carrier-driven mechanism, as local heating around the nanoparticles is minimized under our experimental conditions. These conditions include continuous-wave irradiation, efficient heat transfer from colloidal photocatalysts to aqueous solution, constant stirring of the reaction solutions, and cooling the solutions with a fan.^22,57^ The macroscopic temperature of the reaction solutions increased by 1–2 °C. Second, we anticipate that Co–B nanoparticles have metallic cobalt on their surfaces during the course of the catalyzed reactions. When surface cobalt was oxidized during our storage or handling of the catalysts, it should be reduced to metallic cobalt by hydrazine.^58^ The observed long induction time suggests that this surface modification might happen. Our X-ray photoelectron spectroscopy (XPS) measurements for the Co–B nanoparticles used after the catalyzed reactions confirmed a higher ratio of cobalt metal to cobalt oxides than that for the stock particles (Figure S13). Note that partial oxidation is unavoidable in our XPS measurements due to the exposure of the nanoparticles to air during sample preparation.

### Photocatalysis of Co–B Amorphous Alloyed Nanoparticles for Nitrobenzene Reduction

To explore the photocatalytic mechanisms of Co–B nanoparticles, a photocatalyzed reaction was conducted at varying optical powers and excitation wavelengths. When the incident power increased, the *k*_app_ reached a maximum before decreasing (Figures 1e,f and S9). According to the photocharging mechanism mentioned in the Introduction section, higher optical power increases the rate of generating hot electrons and holes. Given that isopropanol was used in excess as the hole quencher, more hot holes were effectively quenched, and the accumulated electrons raised the Fermi level and facilitated the reduction.^47^ At a much higher optical power, the decrease in *k*_app_ is unexpected. We think that the Fermi level may be so high that the electrons start to fill the metal–adsorbate antibonding states. As a result, the adsorption of nitrobenzene on cobalt was reduced and the overall photocatalytic activity dropped. Further experiments, such as redox titrations to calculate the number of accumulated electrons under photocharging,^47^ or utilizing the Nernst equation at photostationary states to determine the nanoparticles’ reduction potentials, could potentially be pursued in the future to estimate the Fermi level of the photocatalysts.^59^ Another possibility is that the products of the hole-quenching process may be produced faster at high optical power, and they start to poison the catalysts.

**Figure 2:**
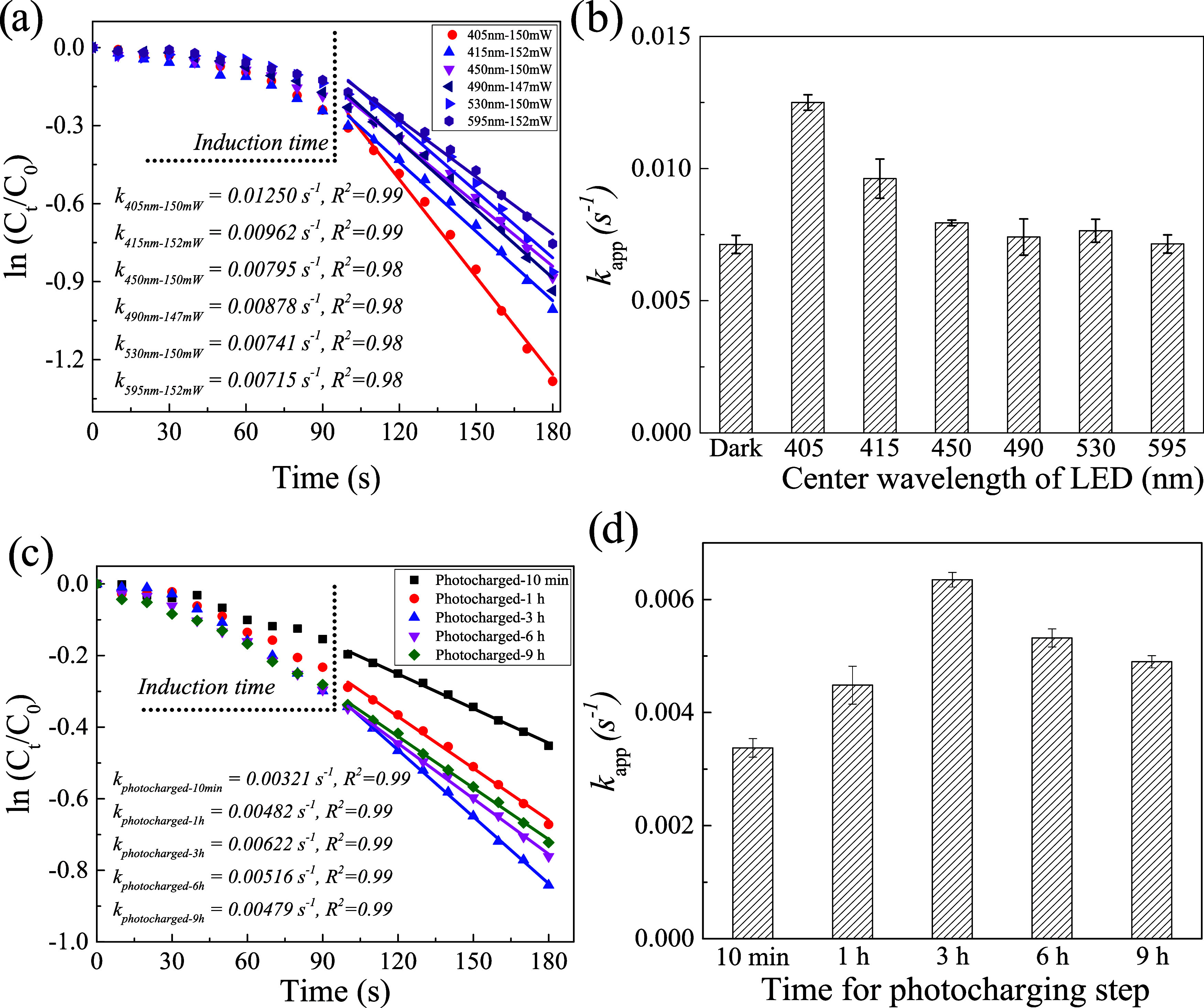
Photocatalytic performance under different excitation wavelengths and evidence of photocharging mechanism of Co–B nanoparticle photocatalysts. (a) Kinetic analysis for nitrobenzene reduction by N_2_H_4_ with Co–B nanoparticles and various excitation wavelengths within a comparable incident power. The linear fits follow a pseudo-first-order process for nitrobenzene. (b) Apparent reaction rate constants (*k*_app_) extracted from (a). (c) Reaction kinetics for nitrobenzene reduction with photocharged Co–B nanoparticles after separating the photocharging from catalysis steps. (d) *k*_app_ extracted from (c). All error bars represent one standard deviation of the mean.

When changing the wavelengths of the irradiated photons, we were aware that the quantum yield of the reaction at different wavelengths is ideal for studying reaction mechanisms.^40^ However, we could not measure the accurate number of photons absorbed by the catalysts because our home-built setup does not allow us to measure the accurate optical power before and after the cuvette (Figure 1d). To have comparable kinetics across all excitation wavelengths, we set the optical power to around 150 mW for all wavelengths. Figure 2b shows that the *k*_app_ values are larger under 405 and 415 nm excitations, but the other *k*_app_ values under longer wavelength excitations are comparable, within experimental uncertainty, to the *k*_app_ under dark conditions. This trend cannot be explained by the absorption curve (Figure 1a) of Co–B nanoparticles or by the small difference in incident photons at different wavelengths. Our interpretation is that only the 405 or 415 nm photons generate holes that are deep enough to be quenched by isopropanol, and this step establishes the observed photocatalysis. This interpretation can be further clarified by conducting more systematic experiments with other hole scavengers and analyzing the corresponding oxidized products. This trend aligns with the *sp-d* interband transitions of cobalt metal in the 345–4100 nm region.^45,60,61^ It is noted that the LSPR of Co nanoparticles has a range of 190–350 nm and a peak at 270 nm, and our experiments have no access to this region.^61,62^

To confirm photocharging process in the photocatalytic mechanism, the photocharging step was separated from the catalytic step.^47^ The Co–B nanoparticles in stock ethanol solution were first charged under a certain irradiation time of a 405 nm LED (450 mW), and the ethanol solvent played the role of the hot-hole scavenger. The charged particles were added to the reaction solutions (more details in the Experimental Methods), and the *k*_app_ was measured. As expected, the *k*_app_ first increased for a longer charging time due to the increase of accumulated electrons for catalyzing the reaction (Figures 2c,2d and S11). As for longer charging time, 6 or 9 h, the photocatalytic activities dropped. We speculate that the oxidized products of the hole-scavenging step may have enough time to adsorb onto the catalysts and block their active sites. This trend is also in alignment with the power dependence of *k*_app_ in Figure 1f. The photocatalysts reduced their activity after absorbing a large number of photons, either from high photon flux or prolonged irradiation conditions.

### Comparison of Photocatalytic Activities of Au, Pd, and Co–B Nanoparticles

**Figure 3:**
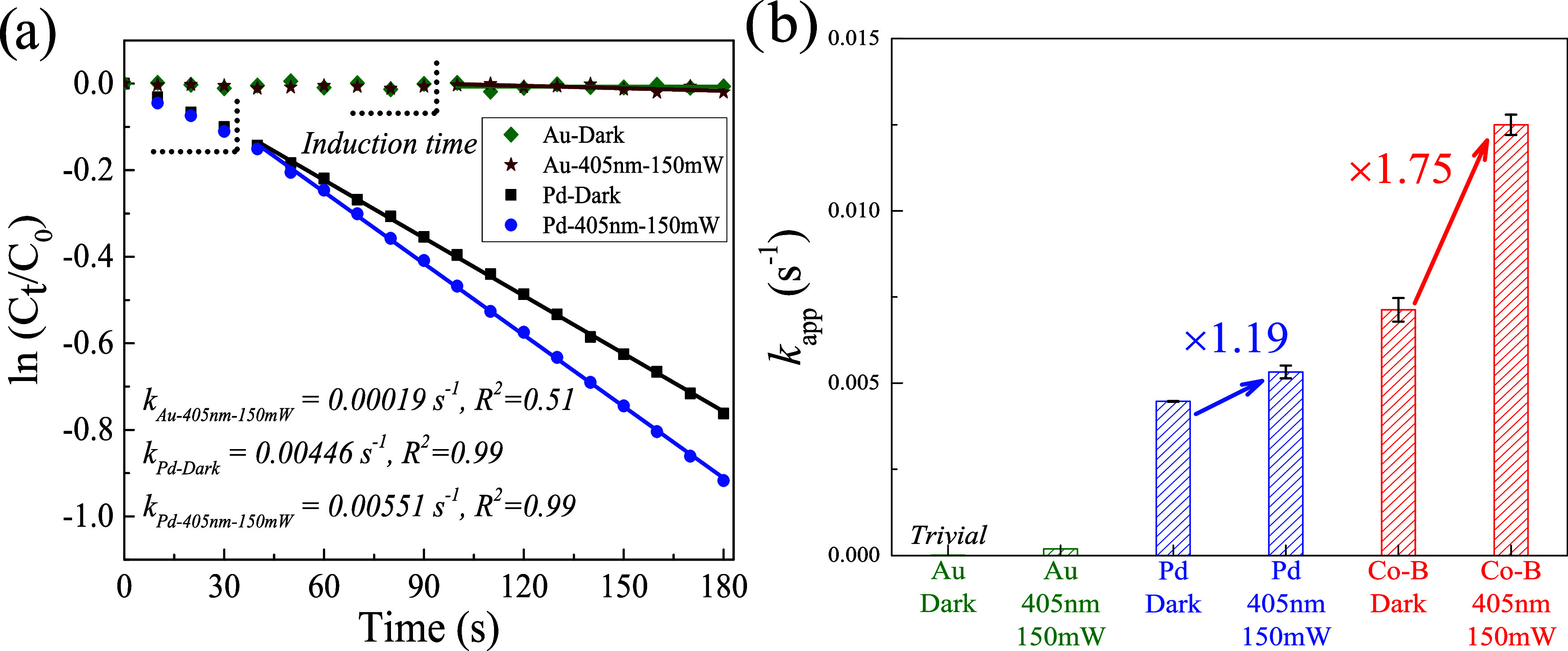
Photocatalytic performance of three metallic nanoparticle photocatalysts. (a) Reaction kinetics for nitrobenzene reduction under typical nonirradiation and irradiation conditions (405 nm LED, 150 mW) with Au and Pd nanoparticles. (b) Comparison of apparent rate constants (*k*_app_) extracted from (a) and Figure 2b. Error bars represent one standard deviation of the mean.

Au nanoparticles showed almost no noticeable catalytic activities under the dark or irradiation conditions within the 3 min time frame in which we compared the catalytic performance of the three nanoparticles. Attempting to extract the *k*_app_ yields a low regression coefficient (Figures 3a and S12). However, a 20% conversion of nitrobenzene was observed when the radiation time was extended to 5 h. Due to these reasons, the photocatalytic enhancement factor (described in the Introduction section, the ratio of *k*_app_ under 405 nm irradiation to *k*_app_ under nonirradiation) for Au nanoparticles is undetermined. Our conclusion is that the particles have the lowest activity for the studied reaction.

Pd nanoparticles offered higher catalytic activities, and the induction time was shortened to 30 s to get better linear fits for *k*_app_ extraction (Figures 3a and S12). This observation is consistent with *p*-nitrophenol reduction under nonirradiation conditions, whereas Pd nanoparticles embedded in spherical polyelectrolyte brushes have lower reaction activation barriers than Au nanoparticles.^52^ In another *p*-nitrophenol reduction by dendrimer encapsulated nanoparticles under nonirradiation conditions, Pd nanoparticles offer higher *k*_app_ than Au nanoparticles because *p*-nitrophenol adsorbs stronger to the Pd surface.^63^ The explanation for this result was the d-band center of Pd is higher than that of Au.^63^ This result can be visualized in Scheme 1. However, we cannot entirely compare the catalytic activities across the Au, Pd, and Co–B nanoparticles under our nonirradiation conditions because they have different morphology, surface area, and capping ligands. Evidently, the Co–B nanoparticles have a less uniform size distribution than the other two nanoparticles (Figures S2 and S3). More importantly, the catalyst load (concentration of metals in reaction solutions) of Pd nanoparticles was around 8.7 times higher than Au nanoparticles and 27 times lower than Co–B counterparts (see details in the Supporting Information), but their catalytic activities (experimental *k*_app_) do not reflect this loading ratio. According to the d-band theory (Scheme 1) and recent density functional calculations, nitrobenzene has a stronger binding to Co than Pd and Au.^64^ However, the calculations also pointed out that the strong binding of either the reactant or intermediate to the metals could be the trade-off in favoring the reduction of reactant to NO-phenyl or not favoring the reduction of OH intermediate to H_2_O. Overall, Co metal has a slightly better activity for nitrobenzene reduction than Au, but Pd has the highest activity for the best balance of the rates of these two elementary steps (the volcano trade-off).^64^ That prediction agrees with our observed *k*_app_, whereas Au nanoparticles have almost no activities and Pd nanoparticles have the highest activities after considering the catalyst load.

As mentioned in the Introduction section, the photocatalytic enhancement factors can mitigate the morphological difference. The Co–B nanoparticles offer a higher photocatalytic enhancement factor (Figure 3b). Our explanation is Co has a stronger binding to the reactant (Scheme 1), which facilitates the electron transfer needed for the reduction reaction. As it is known that the initial reduction of NO_2_-phenyl to NO-phenyl on the metals is often the rate-determining step of the reaction,^64^ we speculate that either the hot electrons or the accumulated electrons due to photocharging catalyze this important step. Another explanation for the higher photocatalytic enhancement for Co–B nanoparticles is that the hot holes in Co–B nanoparticles were quenched by the hole scavenger more efficiently than those in Pd or Au nanoparticles due to the stronger binding between Co and the hole scavenger. This could lead to a stronger photocharging effect, and eventually a higher photocatalytic enhancement. At this point, we can conclude that the catalytic trend observed under nonirradiation conditions (Pd > Co–B > Au) follows the predicted volcano trade-off.^64^ However, the trend of photocatalytic activity (Co–B > Pd > Au) is different as the catalytic pathway is expected to be modified. This new trend indicates that the initial reduction of NO_2_-phenyl to NO-phenyl (favored by the strong metal–adsorbate binding) is better catalyzed by the photocatalysts than the reduction of the OH intermediate to H_2_O (unfavored by the strong metal–adsorbate binding).

## Conclusions

Utilizing interband transitions for photocatalysis can be very applicable to non-noble metallic nanoparticles. We demonstrated that the d-band structures and interband transitions are critical factors in predicting photocatalytic activities of metallic nanoparticles when our study transitions from noble to non-noble metals. When moving to the left of the periodic table, the stronger binding of reactants to the metals can facilitate the hot-carrier transfer and enhance the photocatalytic activity.

## Supporting Information Available

TEM images, DLS, and PXRD of three metallic nanoparticles (Figures S1–S4); UV–vis spectra of standard solutions and control reactions (Figures S5 and S6); qualitative analysis of the reduction products by UV–vis and NMR spectroscopies (Figures S7 and S8); *in situ* UV–vis spectroscopy for kinetics analysis of reduction reactions with different nanoparticles (Figures S9–S12); and surface oxidation analysis of Co–B nanoparticles before and after the catalyzed reactions (Figure S13) (PDF)
