# Supplementary material for: From Precious to Earth-Abundant Metallic Nanoparticles: A Trend of Interband Transitions in Photocatalyzed Nitrobenzene Reduction
Source: J Phys Chem C Nanomater Interfaces. 2024 Aug 22;128(35):14674–82. doi: 10.1021/acs.jpcc.4c03940 (PMC11382268; doi:10.1021/acs.jpcc.4c03940)
Supplement: Supplementary file 1 — jp4c03940_si_001.pdf [file jp4c03940_si_001.pdf]

Supporting Information

From Precious to Earth-Abundant Metallic Nanoparticles: A Trend of Interband Transitions in Photocatalyzed Nitrobenzene Reduction

Pin Lyu,[1,2,*] Lauren Hoffman,[2] Daniel Valenzuela Cahua,[1] and Son C. Nguyen[1,*]

[1]Department of Chemistry and Biochemistry, University of California Merced, 5200 North Lake Road, Merced, California 95343, United States.

[2]Department of Chemistry and Biochemistry, University of North Carolina Asheville, 1 University Heights, Asheville, North Carolina 28804, United States.

*Corresponding Author: plyu@unca.edu son@ucmerced.edu

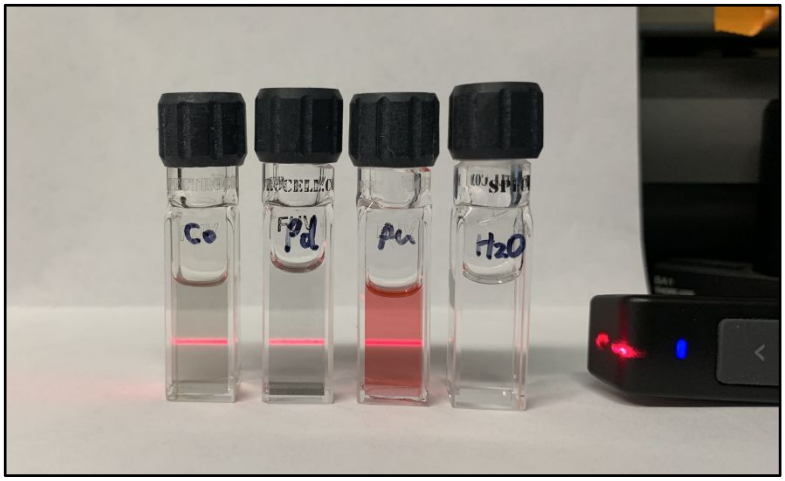

Figure S1. Image showing colloidal form of metallic nanoparticle photocatalysts. From left to right: Co-B nanoparticles in ethanol, Pd nanoparticles in water, Au nanoparticles in CTAC solution, and H_2O as a reference.

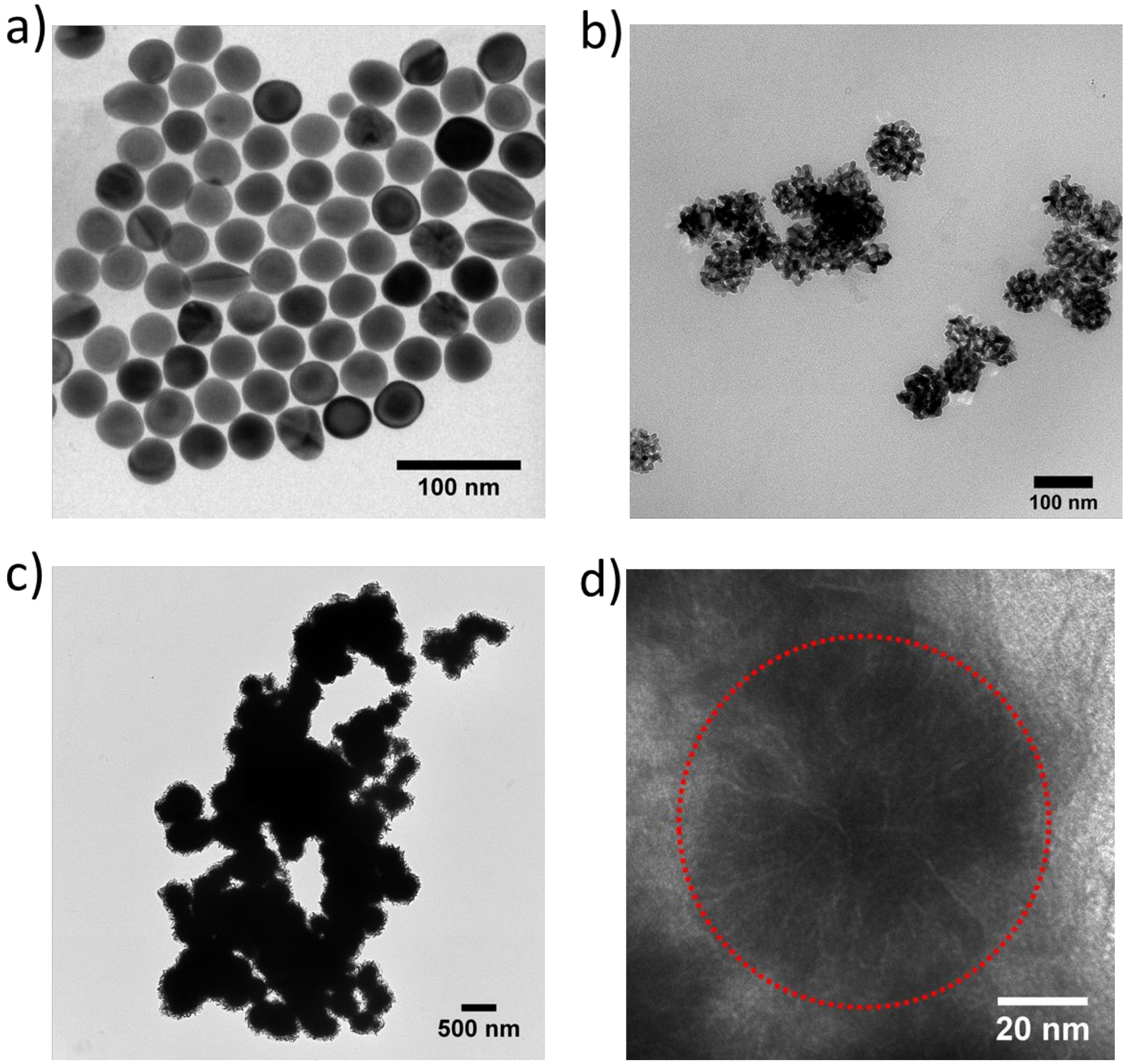

Figure S2. TEM images of (a) Au, (b) Pd, (c) Co-B nanoparticles and (d) a representative Co-B nanoparticle with amorphous structure. The average diameters of Au, Pd, and Co-B nanoparticles are 38 ± 2, 68 ± 12, and 83 ± 22 nm, respectively. Note that the aggregations of Co-B nanoparticles shown in panel c were excluded from size determination.

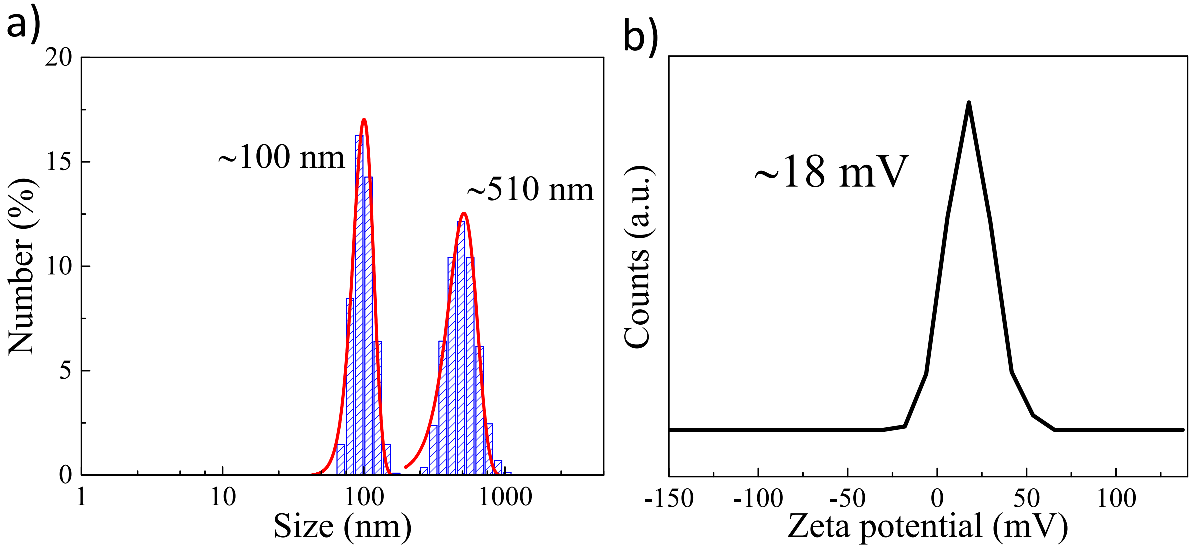

Figure S3. Size distribution and zeta potential of Co-B nanoparticles stock solution from DLS measurements.

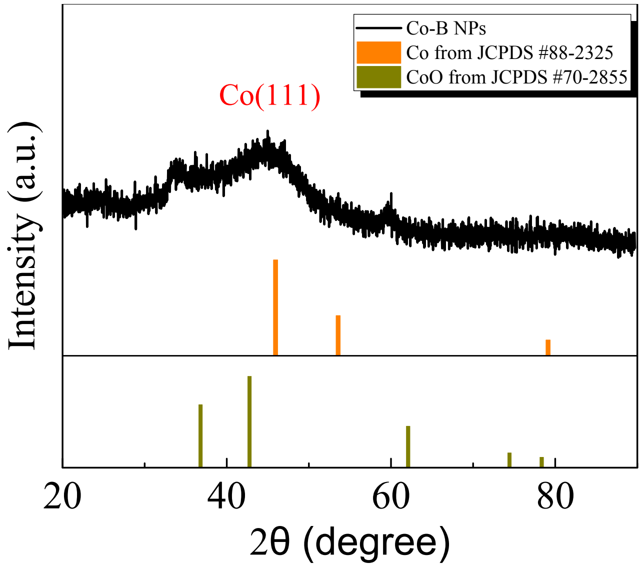

Figure S4. PXRD patterns of Co-B nanoparticles. The JCPDS (Joint Committee on Powder Diffraction Standards) cards were retrieved from X'Pert HighScore data analysis software. The broadening of the Co(111) peak indicates the overall amorphous structure but with small regions of crystalline, which is agreed with previous observations.[1, 2]

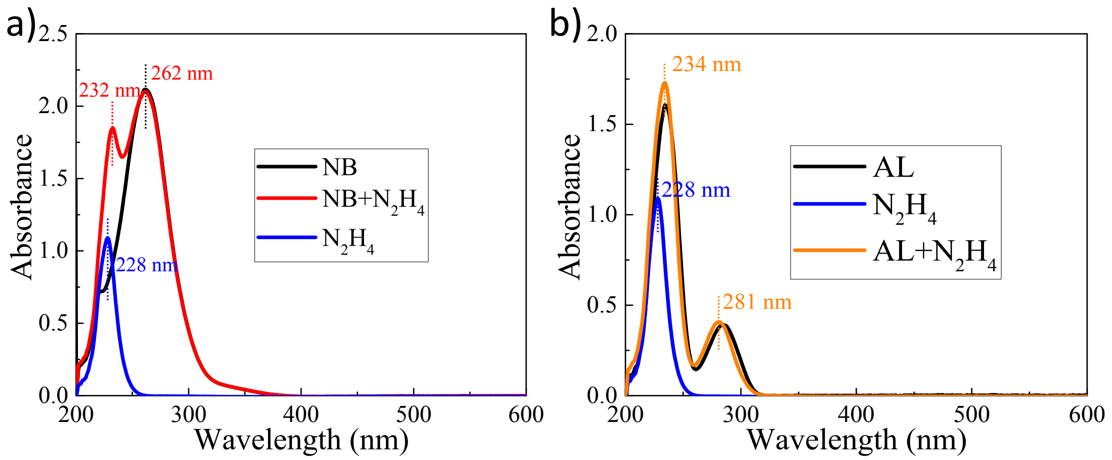

Figure S5. UV-vis spectra of standard solutions of reactants or products in the studied nitrobenzene reduction. (a) Nitrobenzene (NB), hydrazine (N_2H_4), and their mixture. (b) Aniline (AL), hydrazine (N_2H_4), and their mixture. The concentrations of the standard compounds are similar to those in typical reaction conditions.

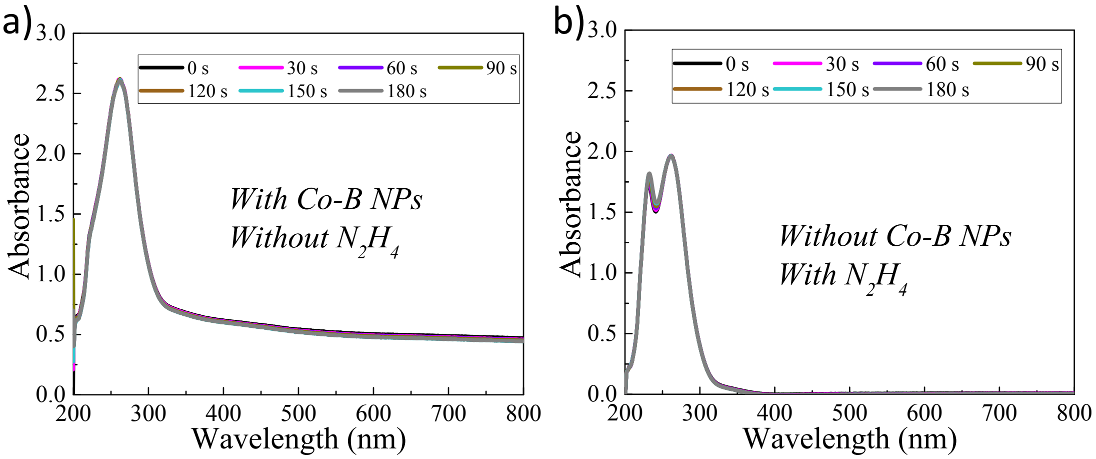

Figure S6. UV-vis spectra of control reaction solutions under dark conditions. (a) with Co-B nanoparticles but not reducing agent N_2H_4, and (b) with N_2H_4 but not Co-B nanoparticles. Other conditions are consistent with those of typical reaction conditions.

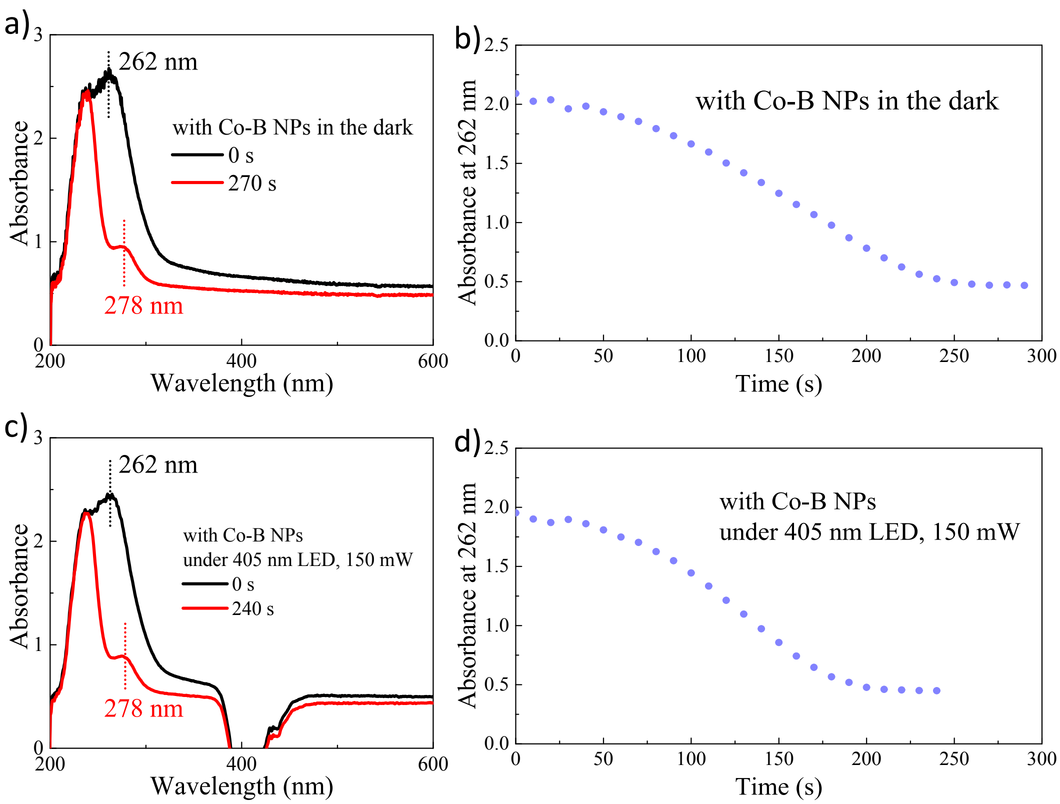

Figure S7. (a and b) UV-Vis spectra and kinetic trace of nitrobenzene (at 262 nm, background subtracted) of a typical reaction solution with Co-B nanoparticles under non-irradiation conditions. (c and d) similar UV-Vis spectra and kinetic trace for the same reaction under typical photocatalyzed conditions with 150 mW incident power of a 405 nm LED. The peak at 278 nm indicates the formation of aniline product. The strong bleach at 400 nm region in panel c comes from the leaking of the 405 nm light to the UV-Vis detector.

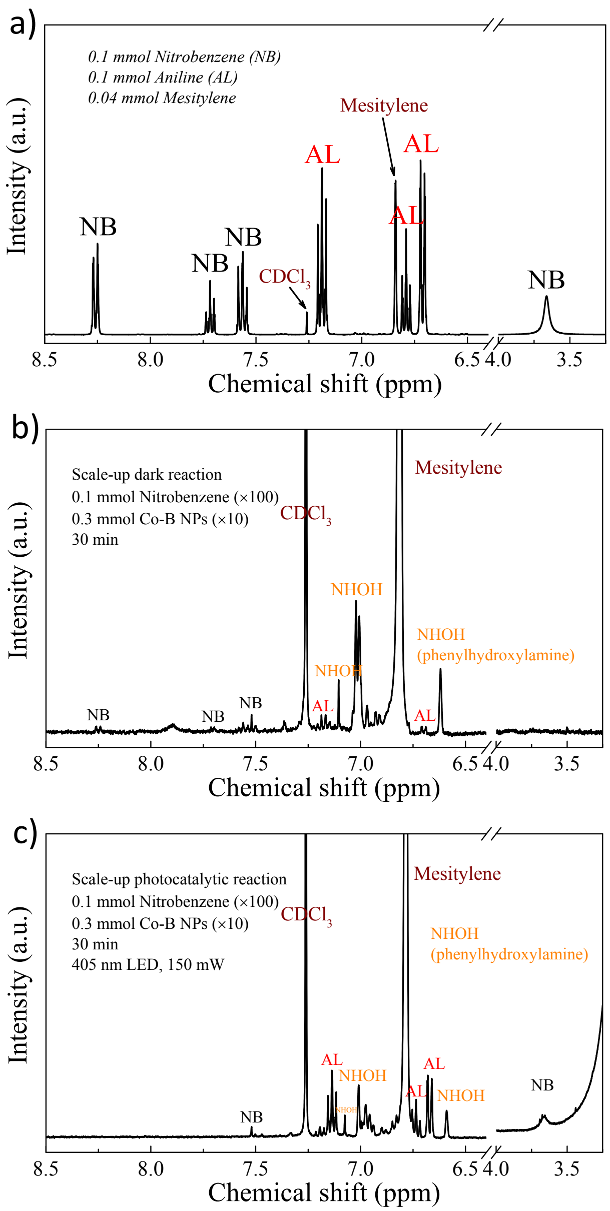

Figure S8. NMR spectra of (a) standard solution of reactants and products in nitrobenzene reduction, (b) scale-up non-irradiation reaction, and (c) scale-up photocatalyzed reaction. The typical reaction was scaled up for better NMR signal. The amount of nitrobenzene was scaled up to 100 times, N_2H_4 to 20 times, and Co-B nanoparticles to 10 times as compared to the typical reaction conditions. Mesitylene and deuterated chloroform were used as the internal standard and extraction solvent. The detection of the reaction intermediate (NHOH, phenylhydroxylamine) and reaction product (AL, aniline) confirmed the electron- and proton- transfer process from nanoparticles to reactants. Due to the multistep mechanisms involved and rapid reduction time, we only monitor the reactant conversion to extract the reaction rate constants rather than the product yield.

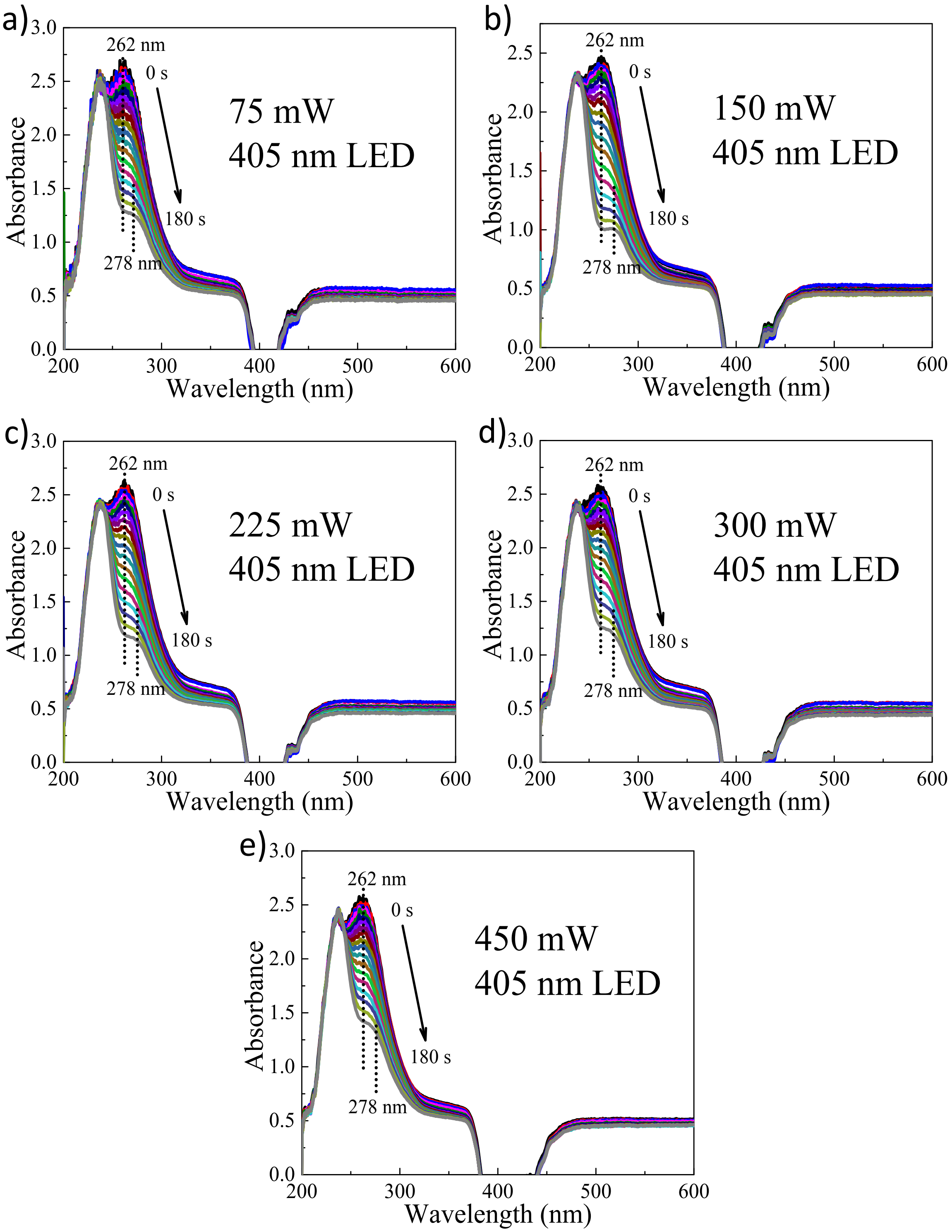

Figure S9. UV-Vis spectra of reaction solutions with Co-B nanoparticle photocatalysts under typical photocatalyzed conditions. The optical power of the LED (centered at 405 nm, FWHM=12 nm) was adjusted from 75 to 450 mW. The reaction rate constants were extracted and plotted in Figure 1e and 1f in the main text. The strong bleach (negative absorbance) at 400 nm region comes from the leaking of the 405 nm light to the UV-Vis detector.

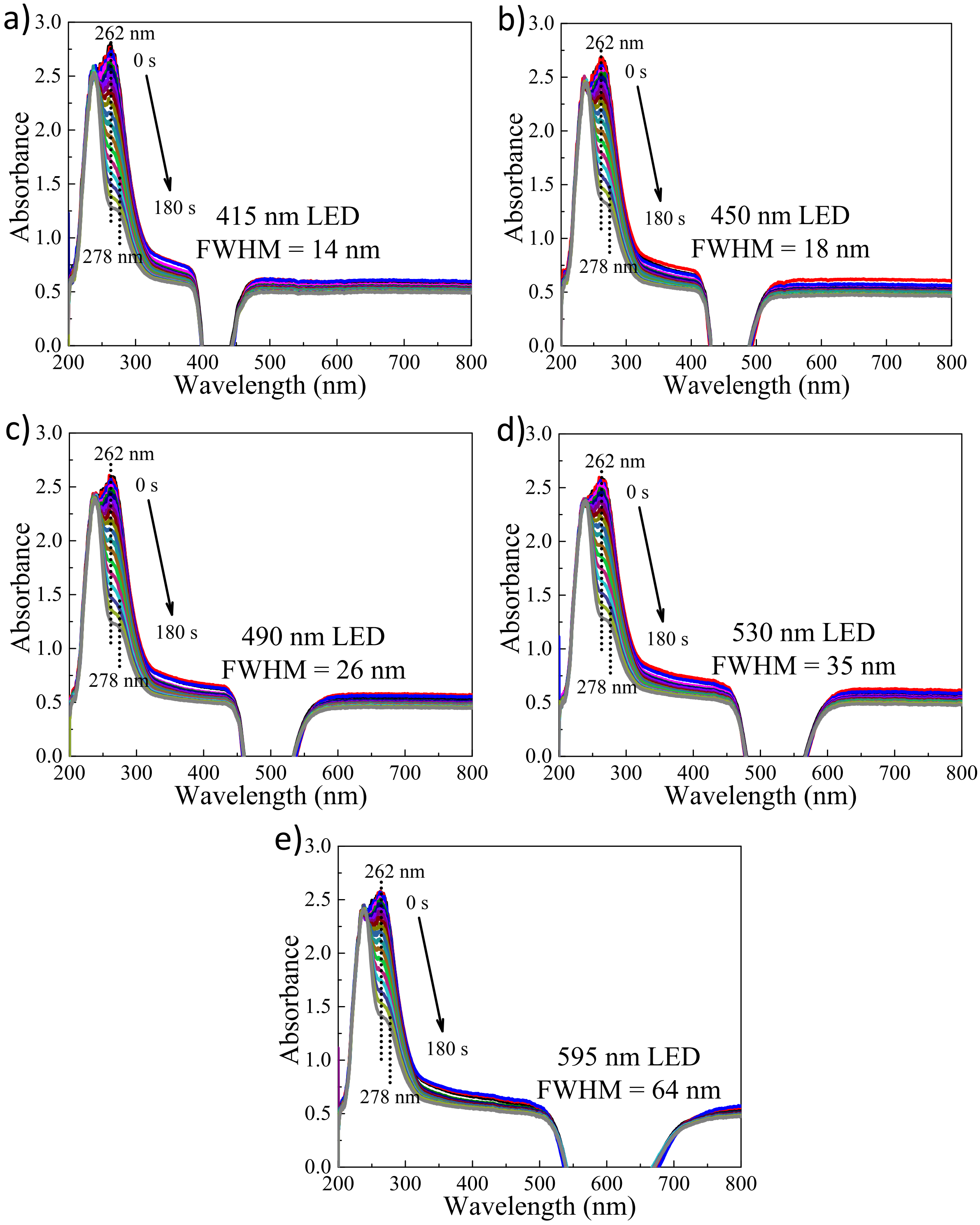

Figure S10. UV-Vis spectra of reaction solutions with Co-B nanoparticle photocatalysts under photocatalyzed conditions with various excitation wavelengths. All the LEDs were adjusted to an incident power of about 150 mW. The reaction rate constants were extracted and plotted in Figure 2b in the main text. The strong bleach (negative absorbance) comes from the leaking of the excitation light to the UV-Vis detector.

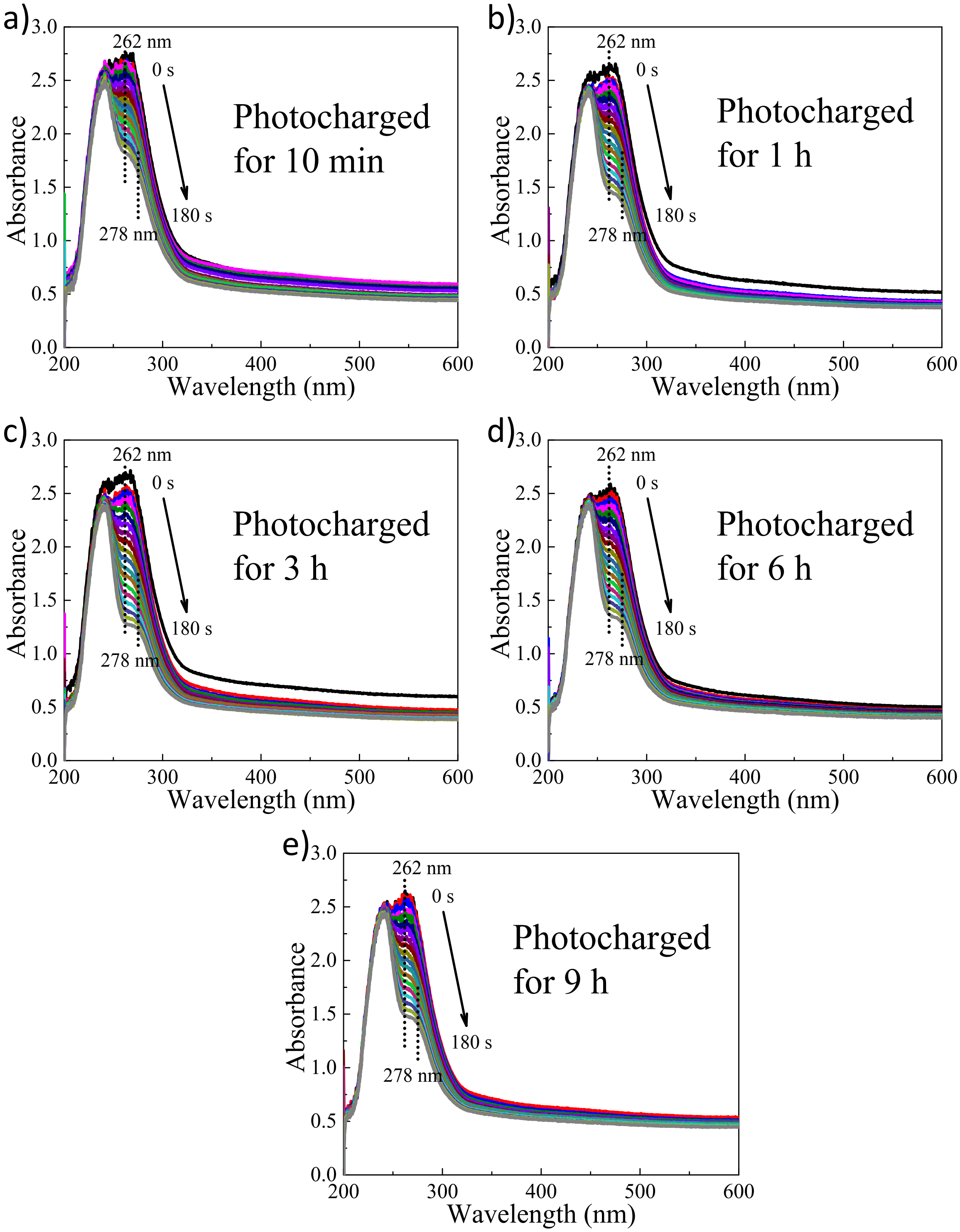

Figure S11. UV-Vis spectra of reaction solutions with Co-B nanoparticles after separating the photo-charging step from the catalysis step. Co-B nanoparticles were photo-charged by a 405 nm LED (450 mW, FWHM=12 nm) with different charging time. The reaction rate constants were extracted and plotted in Figures 2c and 2d in the main text. See the charging conditions in the experimental section of this SI.

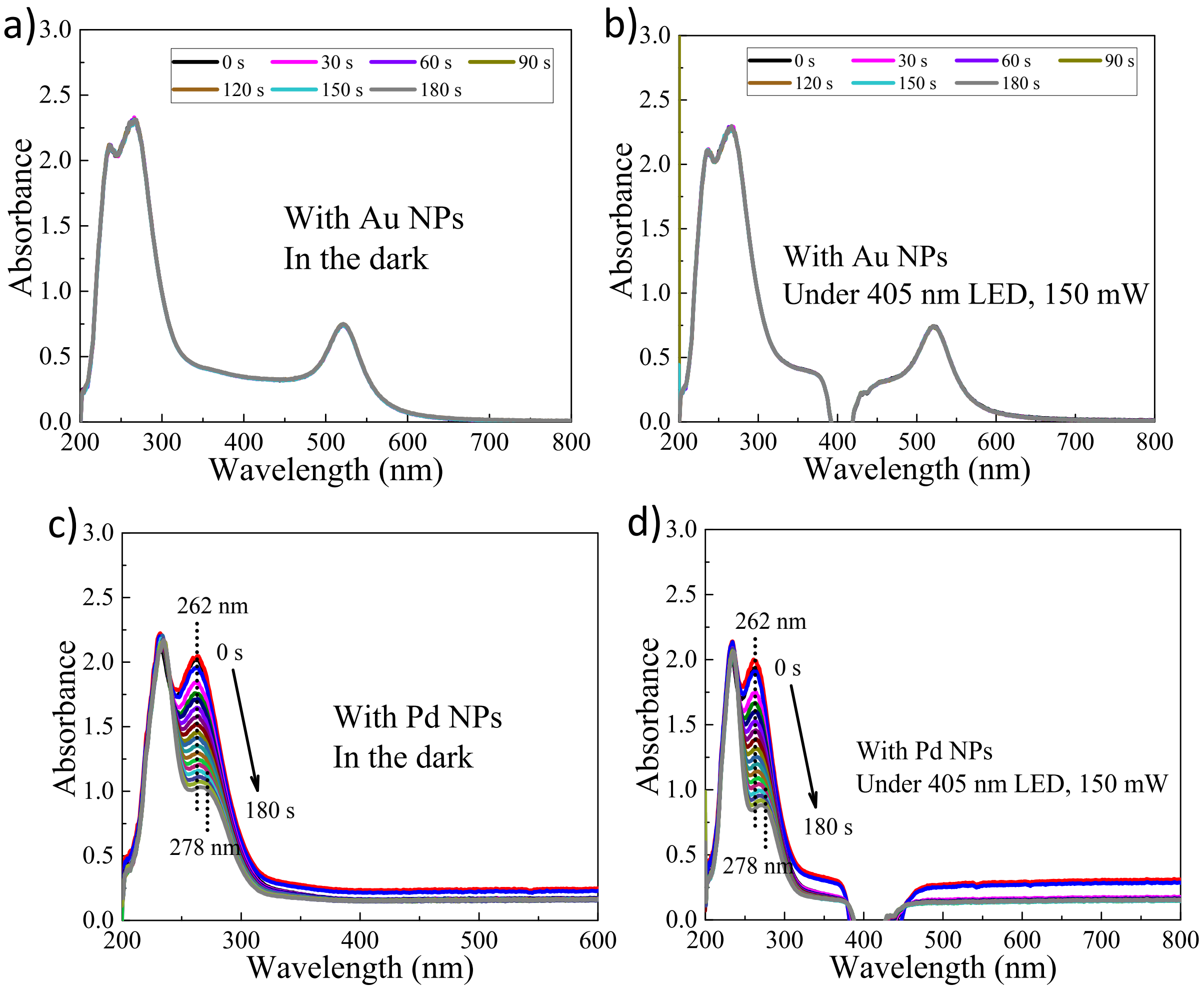

Figure S12. UV-Vis spectra of reaction solutions with Au nanoparticles and Pd nanoparticles under typical non-irradiation and photocatalyzed conditions. (a) Reaction with Au nanoparticles in the dark conditions, (b) Reaction with Au nanoparticles under 150 mW incident power of a 405 nm LED, (c) Reaction with Pd nanoparticles in the dark, and (d) Reaction with Pd nanoparticles under 150 mW incident power of a 405 nm LED. The kinetic data were retrieved and plotted in Figure 3a in the main text.

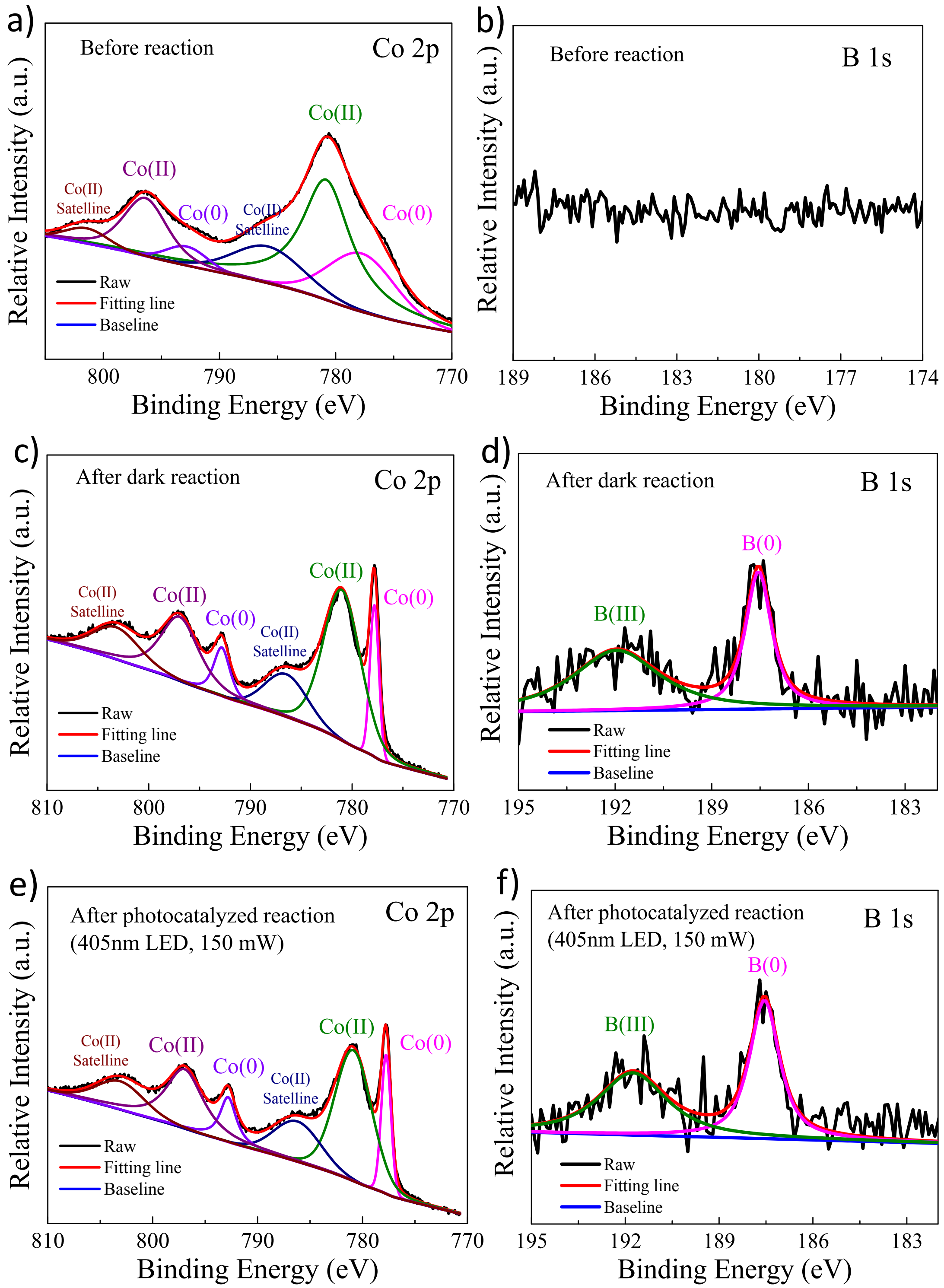

Figure S13. XPS analysis of Co-B nanoparticle catalysts (a, b) before reduction reaction, (c, d) after reduction reaction under non-irradiation conditions, and (e, f) after a typical photocatalyzed reaction (405 nm LED, 150 mW of incident power).

XPS interpretation for Figure S13: Due to the active nature of cobalt, the surfaces of Co-B nanoparticles were expected to be partially oxidized as soon as they were exposed to air during

sample preparation for XPS analysis.[3] The spectral assignment and deconvolution analysis were based on the XPS handbook and literature.[4-9] Starting with the stock Co-B nanoparticles (Figure S13 a, b), the peaks at 778.1 eV for Co 2p3/2 and 792.9 eV for Co 2p1/2 indicate the metallic state of cobalt in the nanoparticles, while the Co^{2+} species (cobalt oxides) were also observed at a higher binding energy. This result confirmed the metallic form of the stock Co-B nanoparticles with some oxides on their surfaces, which was in consistent with other reports.[5-7] However, no obvious peak of boron was observed. We speculate that the cover of cobalt oxide layers might block the boron detection. Moving to the XPS spectra of Co-B nanoparticles used after the non-irradiation and photocatalyzed reactions (Figure S13, c-f), a sharp peak of metallic Co at around 777.8 eV, separated from Co^{2+} peaks, was observed in both samples.[5] This metallic Co peak was shifted about 0.3 eV compared to the Co peak before the reaction and indicated the electron transfer from the metallic Co to elemental B.[5] For boron, the peak at around 187.5 eV in B 1s spectra (Figure S13, d and f) was corresponding to unoxidized boron on the surface of Co-B nanoparticles, and there were some boron oxides formed due to sample preparation with air exposure.[9] Lastly, the percentage of metallic Co in both samples after reactions was estimated by peak area analysis to be 16 % after the dark reaction and 18 % after the photocatalyzed reaction, which were quite close and showed that the numeric change of active sites was not the major contribution to the difference of catalytic activity in those two conditions.

References:

(1) Zhu, Z.; Ma, J.; Xu, L.; Xu, L.; Li, H.; Li, H. Facile Synthesis of Co–B Amorphous Alloy in Uniform Spherical Nanoparticles with Enhanced Catalytic Properties. *ACS. Catal.* **2012,** *2,* 2119-2125.

(2) Li, H.; Wu, Y.; Luo, H.; Wang, M.; Xu, Y. Liquid phase hydrogenation of acetonitrile to ethylamine over the Co-B amorphous alloy catalyst. *J. Catal.* **2003,** *214,* 15-25.

(3) Tompkins, H. G.; Augis, J. A. The Oxidation of Cobalt in Air from Room Temperature to 467°C. *Oxid. Met.* **1981,** *16,* 355-369.

(4) Chastain, J.; King Jr, R. C. *Handbook of X-ray Photoelectron Spectroscopy*. Perkin-Elmer, USA: 1992; p 261.

(5) Jiang, B.; Song, H.; Kang, Y.; Wang, S.; Wang, Q.; Zhou, X.; Kani, K.; Guo, Y.; Ye, J.; Li, H.; Sakka, Y.; Henzie, J.; Yusuke, Y. A Mesoporous Non-Precious Metal Boride System: Synthesis of Mesoporous Cobalt Boride by Strictly Controlled Chemical Reduction. *Chem. Sci.* **2020,** *11,* 791-796.

(6) Masa, J.; Weide, P.; Peeters, D.; Sinev, I.; Xia, W.; Sun, Z.; Somsen, C.; Muhler, M.; Schuhmann, W. Amorphous Cobalt Boride (Co_2B) as a Highly Efficient Nonprecious Catalyst for Electrochemical Water Splitting: Oxygen and Hydrogen Evolution. *Adv. Energy Mater.* **2016,** *6,* 1502313.

(7) Fernandes, R.; Patel, N.; Kothari, D. C.; Miotello, A. Harvesting Clean Energy Through H_2 Production Using Cobalt-Boride-Based Nanocatalyst. In *Advanced Nanomaterials in Biomedical, Sensor and Energy Applications*, Chattopadhyay, J.; Srivastava, R., Eds. Springer Singapore: Singapore, 2017; pp 35-56.

(8) Popat, Y.; Orlandi, M.; Gupta, S.; Bazzanella, N.; Pillai, S.; Patel, M. K.; Miotello, A.; Patel, N. Morphological and Elemental Investigations on Co–Fe–B–O Thin Films Deposited by Pulsed Laser Deposition for Alkaline Water Oxidation: Charge Exchange Efficiency as the Prevailing Factor in Comparison with the Adsorption Process. *Catal. Lett.* **2022,** *152,* 438-451.

(9) Kang, Y.; Jiang, B.; Yang, J.; Wan, Z.; Na, J.; Li, Q.; Li, H.; Henzie, J.; Sakka, Y.; Yamauchi, Y.; Asahi, T. Amorphous Alloy Architectures in Pore Walls: Mesoporous Amorphous NiCoB Alloy Spheres with Controlled Compositions via a Chemical Reduction. *ACS Nano* **2020,** *14,* 17224-17232.
